# mSphere of Influence: the View from the Microbiologists of the Future

**DOI:** 10.1128/mSphere.00348-19

**Published:** 2019-06-19

**Authors:** Aaron Mitchell, Ira Blader, Patricia Bradford, Sarah D’Orazio, W. Paul Duprex, Craig D. Ellermeier, Ana Fernandez-Sesma, Michael J. Imperiale, Katherine McMahon, Marcela F. Pasetti, Susannah Tringe

**Affiliations:** a Carnegie Mellon University, Pittsburgh, Pennsylvania, USA; b University at Buffalo, Buffalo, New York, USA; c Antimicrobial Development Specialists, LLC, Nyack, New York, USA; d University of Kentucky, Lexington, Kentucky, USA; e University of Pittsburgh School of Medicine, Pittsburgh, Pennsylvania, USA; f University of Iowa, Iowa City, Iowa, USA; g Icahn School of Medicine at Mount Sinai, New York, New York, USA; h University of Michigan Medical School, Ann Arbor, Michigan, USA; i University of Wisconsin—Madison, Madison, Wisconsin, USA; j University of Maryland School of Medicine, Baltimore, Maryland, USA; k Department of Energy, Joint Genome Institute, Walnut Creek, California, USA

## EDITORIAL

Which ideas will shape the microbiology of the future? How will they do so? In our new series of “mSphere of Influence” commentaries, we ask rising young microbiologists to tell us just that. We focus on these young scientists because they are uniquely capable of seeing the scientific literature in the context of their own 21st-century education and training.

Our specific request is for our young colleagues to identify an article or a series of articles that have strongly influenced their thinking and to explain how the paper(s) helped propel their careers and scientific viewpoints. We ask for brevity rather than an in-depth scholarly discussion so that our contributors will distill the content and impact of their chosen article into a succinct message. We see these commentaries as reflective journal clubs, with a presentation that captures both the essence of the article and its key influential features.

These commentaries will be valuable to our community in several ways. First, they will provide a view of how ideas influence and excite people who are entering microbiology. We might all use that information to inspire and foster the curiosity of our trainees about our field and profession. Second, they offer a deeper personal perspective on the impact of a publication than could be gleaned from the number of times it has been cited, tweeted, or liked. Third, they provide an essential reading list with unique annotation that conveys the articles’ contributions and sphere of influence. Our hope is that you will join us in reading not only the commentaries but the original research articles that have inspired the future of microbiology.

